# 

**Published:** 1836-04-01

**Authors:** 


### BIBLIOGRAPHICAL RECORD;

OR,

*Works received for Review since last Quarter.*

1. Anatomie Pathologique du Corps Humain, &c. Par J. CRUVEILHIER, M.D. Livraison No. 21, folio. Paris et Londres, Bailliere, Dec. 1835.

2. Traité complet de l'Anatomie de l'Homme, comprenant la Médecine Operatoire. Par le Docteur BOURGERY. Avec Planches, &c. 27 Livraison, 1835.

3. Illustrations of the Comparative Anatomy of the Nervous System. By JOSEPH SWAN. Part I. price 10s. 6d. 4to, pp. 21. Seven Plates, with Letter-press Descriptions. Longman and Co. December, 1835.

4. A Practical Treatise on Urethritis and Syphilis:—Including Observations on the Power of the Menstruous Fluid and of Leucorrhœa, &c. to produce Urethritis; with a Variety of Examples, Experiments, Remedies, and Cures. To which is added, the Proposal of a Substitute for Mercury. By W. H. JUDD, M.R.C.S. Surgeon in His Majesty's Fusileer Guards. Octavo, pp. 568, with coloured Plates. Highley, Fleet-street, Dec. 1835.

5. Introductory Lectures to a Course on Nervous Irritation, Spinal Affections, Distortions of Limbs, Malformations of the Chest, &c. Delivered by J. EVANS RIODORE, Esq. F.L.S. Lecturer on Surgery, &c. Small 8vo, pp. 107. Churchill, Dec. 1835.

6. Sketch of the Medical and Statistical History of Epidemic Fevers in Ireland, from 1798—and on Pestilential Diseases since 1823, with an Appendix, &c. By WILLIAM STOKES, M.D. Octavo, pp. . 1835.

7. A Popular Manual of the Art of preserving Health; embracing the Subjects of Diet, Air, Exercise, Gymnastics, General and Physical Education, &c. By Mr. J. B. DAVIS, Surg. Octavo, pp. 504. Whittaker, 1836.

8. Remarks on the Unity of the Body, as illustrated by some of the more striking Phenomena of Sympathy, both Mental and Corporeal, &c. By GEORGE MACILWAIN, M.R.C.S. Octavo, pp. 294. 1836. Highley, Fleet-street.

9. Rudiments of Pathology. By Dr. FLETCHER. Parts II. *a*, and II. *b*. Octavo, Edinburgh, 1836.

☞ *We consider Dr. Fletcher as, by far, the most original thinker—or at least* WRITER, *of the present day.*

10. The Veterinarian; or Monthly Journal of Veterinary Science, for January and February, 1836 (New Series). Edited by Messrs. DICK, KARKEEK, PERCIVAL, and YOUATT.

11. The ROYAL SOCIETY in the Nineteenth Century; being a Statistical Summary of its Labours during the last 35 years, with many original Tables and Official Documents (never before published), shewing the Constitution of the Society, &c. and a Plan of Reform, &c. By A. B. GRANVILLE, M.D. F.R.S. Octavo, pp. 253. Churchill, London, Feb. 1836.

☞ *This is the result of considerable labour, and interspersed with caustic criticisms and remarks, that will render it a piquant treat to every* F.R.S. *in England.*

12. On Insanity; its Nature, Causes, and Cure. By W. B. NEVILLE, Esq. Octavo, pp. 192. Feb. 1836.

☞ *This is a very scientific compendium, evincing considerable research and sound judgment. There are many original and judicious observations, in this volume, on therapeutics. In our next Number we shall adduce a very interesting extract, respecting large doses of soda in indigestion.*

13. A brief Memoir of Sir William Blizard, F.R.S. &c. By WILLIAM COOKE, M.R.C.S. Octavo, pp. 67.

14. A Letter to the Chairman and Guardians of the Chelmsford Union, relating to Medical Attendance.

---

15. An Essay on the Laryngismus Stridulus, or Croup-like Inspiration of Infants. To which are appended, Illustrations of the General Principles of the Pathology of Nerves, and of the Functions and Diseases of the Par Vagum and its principal Branches. By Hugh Ley, M.D. Physician to the Westminster General Lying-in Hospital, &c. &c. Illustrated with Plates, 1 Vol. 8vo, pp. 480. March, 1836. Churchill, London.

☞ *We regret that this Work came to hand after our Review department was closed. It is one of the most important essays that has appeared in this country during the present century. We shall dedicate two or three extensive articles to its analysis in our succeeding Numbers.*

---

16. A Manual of Medical Jurisprudence and State Medicine, compiled from the latest Authorities, &c. By Michael Ryan, M.D. &c. Second Edition. Octavo, pp. 538. Gilbert, Piper, and Jones. February, 1836.

☞ *The indefatigable author of this Manual has employed great labour and talent in the construction of this work; and a new edition demonstrates the favourable reception which it has met with from the Public.*

---

17. A Letter to the Reform Corporations on the Necessity of electing Medical Coroners. By George Rogerson, Surgeon of Liverpool.

☞ *We have stated our own sentiments on this point in another place.*

---

18. A Treatise on Epidemic Cholera, as observed in the Duane-street Cholera Hospital, New York. By Floyd T. Ferris, Esq. with three beautifully-coloured Plates.

☞ *The disease has now lost all interest in this country, to which it is very unlikely to return.*

19. On the Analysis of the Blood and Urine, in Health and Disease; with Directions for the Analysis of Urinary Calculi. By G. A. Rees. Octavo, pp. 187. Longman and Co. March, 1836.

---

20. A Report on the Medical Management of the Native Jails, throughout the Territories subject to the Governments of Fort William and Agra. To which are added, some Observations on the principal Diseases to which Native Prisoners are liable. By James Hutchinson, A.M. &c. Octavo, pp. 107. Calcutta, 1835.

☞ *This little work will afford very useful materials to the Indian practitioners; but is not calculated to interest the faculty of this country. We shall give an extract or two from the volume in our next Number.*

---

21. New Treatment of Malignant Diseases, and Cancer without Incision. By A. M. Bureaud Reofrey, M.D. Octavo, pp. 78. Longman and Co. 18[illegible]5.

---

22. Education Physique des Jeunes Filles, avant le Marriage. Par A. M. Bureaud Reofrey, M.D. Octavo, pp. 338. Dulau, 1835.

---

23. A Treatise on Pulmonary Consumption. By Robert Little, M.D. Professor of Midwifery, &c. in the Belfast College, &c. Octavo, pp. 160. Longman and Co. March, 1836.

---

24. The Physical and Intellectual Constitution of Man considered. By Edward Meryon, F.R.C.S. 8vo, pp. 240. Smith, Elder, and Co. March, 1836.

☞ *On the Plan of Dr. Pritchard's great work. The essay is very creditable to Mr. Meryon.*

---

27. A Lecture on the Elements of Physical Education. By Louis Frechet. Octavo, pp. 36.

### INDEX.

Abdominal disease, fatal, Dr. Johnson's case of .................. 161
Abscess in bone .................. 7
Abscess, fæcal, case of ............ 567
Absorption and ulceration ........ 446
Academy, royal, of Medicine, memoirs, 517
Adipose tissue, pathology of the .... 340
Advantages of no advantages ...... 495
Affections of the brain ............ 117
Air, introduced into bloodvessels, M. Clot Bey on ........ ........ 518
Aldis's, Dr. cases of pulmonary apoplexy 171
Amaurosis ...................... 53
Anæmia, Dr. Geddes on .......... 172
Aneurism of the thoracic aorta, striking cases of.................... 148
Aneurysm, report on.............. 258
Aneurysm of the post tibial artery .. 258
Aneurysm of the popliteal artery.... 259
Aneurysm of the brachial artery .... 259
Aneurysm of the heart, description of, 349
Aneurysm, clinical report on ...... 577
Aneurysmal varyx, cured by bandage, 261
Aneurysmal varyx, ligature of the brachial artery successful ........ 290
Aneurysmal tumor in the orbit .... 584
Angina pectoris, Dr. Wilson on .... 411
Aorta, case of obliteration of ...... 217
Aponeuroses, pathology of the ...... 339
Apoplexy, simple sanguineous ...... 121
Arachnidan condylopes ............ 90
Arachnoid, spinal, cartilaginous deposits on the .................. 547
Arteries, cessation of pulse in many.. 451
Articular epiphyses, great enlargement of, from rickets............ 182
Atheist, picture of the ............ 400
Atmosphere, foreign bodies in the .. 399
Atrophy of bone.................. 5
Atrophy of brain, Dr. Sims on...... 131
Auscultatory signs of pregnancy .... 220
Auscultatory signs in labour . .... 224
Barry's, Sir David, life and death.... 298
Bedside medicine, Dr. Thorburn's .. 177
Biliary calculi, in lacerated parts of liver 296
Birds, functions and instincts of .... 361
Bladder, puncture of, above pubes .. 166
Bladder, cases of stone in the ...... 197
Bladder, hydatids of the .......... 255
Bladder, extraction of broken catheter from, by the urethra ............ 553
Blood, extravasated alterations of.... 29
Blood, cases of a milky state of...... 229
Bone, hypertrophy of ............ 1
Bone, atrophy of ................ 5
Bone, simple inflammation of ...... 7
Bone, abscess in.................. 7
Bone, caries of ................ 9
Bone, malignant growth of ........ 12
Bone, osteo-sarcoma of............ 12
Bone, medullary sarcoma of ........ 13
Bone, fibrous sarcoma of .......... 14
Bone, cyst-like tumor of .......... 14
Bone, melanosis in................ 14
Bone, hydatids in ................ 15
Bone, strumous caries of .......... 25
Bones of pelvis, fractures of...... . 107
Bostock, Dr. on the composition of calcareous tumors .............. 472
Bouillaud, M. on diseases of the heart, 353
Brachet on the ganglionic nerves .... 57
Brachet, M. on the ganglionic system of nerves ...................... 414
Brachial artery tied for aneurysmal varyx ...................... 200
Brain, suppression of the .......... 66
Brain, influence of, on the heart .... 68
Brain, affections of the ............ 117
Brain, hypertrophy and atrophy of, by Dr. Sims ...................... 117
Brain, cure of ramollissement of .... 117
Brain, hypertrophy and atrophy of, by Dr. Sims .................. 125
Brain, hypertrophy of—enteritis .... 126
Brain, hypertrophy of—extravasation of blood ...................... 126
Brain, hypertrophy of, convulsions, &c. 127
Brain, hypertrophy of—pulmonary tubercles . .. .................. 127
Brain, with apoplectic cysts ........ 128
Brain, atrophy of—cases .......... 131
Brain, Dr. Sims on ramollissement of, 135
Brain, remarkable disease of, Dr. Johnson's case of ....... . .... 202
Brain, pathology of .............. 563
Brain, case of disease of .......... 564
Brain, diseased, state of tongue...... 565
Bridgewater Treatise, Dr. Prout's.... 385
Bright, Dr. on diseased cerebral arteries, 545
Brodie, Sir B. on epulis ............ 278
Brodie, Sir B. on diseases of the rectum, 281
Brodie, Sir B. on corns and bunions.. 585
Bubo, before primary sore.......... 322
Bubo, is the matter from it infectious? 324
Bursæ mucosæ, diseases of ........ 27
Calcareous tumors, chemical composition of ...................... 472
Calculi, large, Mr. Guthrie on the removal of ...................... 287
Calculus in the bladder, cases of .... 197
Cardiac malformation, extreme .... 215
Caries of bone, varieties of ........ 9
Caries of bone, simple ............ 9
Caries of bone, syphilitic .......... 9
Caries of two upper cervical vertebræ, paralysis from.................. 570

Caries of bone, strumous .......... 10
Carswell's, Dr. Illustrations of disease, 1
Carswell's, Dr. Elements of Pathology, 328
Carswell, Dr. on hypertrophy ...... 345
Cartilages, chronic ulceration of .... 23
Catheter broken in bladder, extracted by urethra .. ............... .. 553
Cellular tissue, diffuse inflammation of 335
Cellular tissue, chronic abscess of the, 336
Cellular tissue, cachectic abscess of .. 337
Cellullar tissue, tumors of ........ 338
Cerebral disease—how influenced by hypertrophy of heart............ 507
Cerebral arteries, diseased states of .. 545
Chemistry, in proof of natural religion, 387
Clendinning, Dr. on hydr. of potass.. 241
Climate, Dr. Prout on ............ 395
Clinical instruction, remarks on . .. 276
Clinical report on aneurysm........ 577
Clot Bey on air in the veins, &c. .... 518
Clot Bey on torsion of arteries...... 518
Cock, Mr. on malformations of the internal ear .................... 194
Colon, Dr. Johnson's case of disease of, 184
Colon, extensive adhesions, &c. of .. 470
Colour of snow; why white? ...... 396
Compression for wounded brachial artery in venæsection ............ 552
Constitutional irritation, Mr. Travers, 37
Creation of animals, wisdom of God in, by Mr. Kirby................ 79
Crinoideans..... .... .......... .. 80
Condylopes ...................... 80
Condylopes, power of multiplying in.. 81
Continued fever, report on ........ 266
Continued fever, cases of .......... 267
Continued fever, lesions of ........ 270
Contraction of extremities, from spinal disease .... ................ 547
Convulsions from intestinal irritation, 173
Conwell, Mr. on diseases of the liver.. 371
Cook, Mr. his case of loss of uterus .. 482
Corns and bunions, Sir B. Brodie on.. 585
Coroners' inquests ................ 593
Cranium, case of partial absorption of, 285
Creosote in phthisis .............. 464
Creosote in epilepsy .............. 464
Creosote in neuralgia.............. 464
Creosote in vomiting .............. 465
Creosote in diabetes .............. 466
Creosote in glanders .............. 466
Crusta, genu equini, for epilepsy .... 481
Crusta, genu equini, forms of ...... 481
Crustacean condylopes ............ 82
Crustacean condylopes, mode of casting their crusts ................ 83
Cyclopædia of Anatomy, Parts II., III. 289
Cyclopædia of Anatomy, Part V..... 587
Davies, Dr. his lectures on diseases of the lungs and heart ............ 353
Delirium tremens, remarks on ...... 366
Delirium tremens, case of.......... 459
Delirium tremens, curious case .... 555
Delirium tremens, after injury of head, 556
Delirium tremens, Mr. Tyrrell on .. 557
Deltoid muscle, chronic inflammation of 333
Diagnosis of peritoneal adhesions, observations of ................ 110
Disease, Dr. Carswell's illustrations of, 1
Disease of heart, liver, &c. ........ 588
Diseases of the bursæ mucosæ ...... 27
Diseases of stomach, Mr. Parker on.. 167
Dislocation of os femoris downwards and backwards ... ............ 271
Dropsies, connected with suppressed perspiration, Dr. Osborne on...... 158
Dropsy, from checked perspiration, 159
Dropsy, terminating in profuse diuresis 458
Dry rot—Kyan's patent............ 521
Ear, internal, malformations of...... 194
Ear, death from nitric acid poured into, 562
Earle, Mr. on fractures of the bones of the pelvis .................. 107
Eastern Provincial Medical Association 166
Eczema rubrum—remarkable case of, 573
Education, provincial medical ...... 173
Elliotson, Dr. on creosote.......... 463
Emetic tartar in intermittent fever .. 189
Emplastro-endermic treatment of mania 571
Empyema, curious case of.......... 192
Encysted tumor of neck, cured by seton, 560
Endocarditis, M. Bouillaud on ...... 354
Endocarditis, symptoms of ........ 357
Endocarditis, causes of ............ 357
Enz, Dr. on spinal diseases ........ 523
Epidemic scarlet fever, Dr. Sandwith on 175
Epidemic scarlatina maligna in Yorkshire, Mr. Howard on .......... 292
Epiglottis, symptoms of wound of .. 563
Epilepsy, new remedy in .......... 480
Epiphyses, great enlargement of, in rickets.......................... 182
Epulis, Sir B. Brodie on .......... 278
Epulis, characters of .............. 278
Epulis, treatment of .............. 279
Erectile tumors, M. Lallemand on .. 504
Erectile tumors, various cases of ... 505
Excision of diseased joints.......... 25
Excision of cornea, for staphyloma .. 543
Fæcal abscess ....... ............ 567
Fatty alteration of muscles, case of .. 254
Fever, action of heart and arteries in.. 407
Fever, topical causes of............ 408
Flannel in hot climates ............ 467
Fœtal kidney, Dr. Lee on the........ 453
Fishes .......................... 91
Fishes, difficulty of classifying ...... 92
Fishing frog ........ .......... 93
Fracture, ununited, of humerus, cured by excision .................. 561
Fractured cervix femoris, cases of .. 103
Fractured patella, odd union of...... 273
Fractured external condyle of femur.. 273
Fractured skull, with depression .... 274

Fractured humerus, from muscular exertion ... 274
Fractures of the extremities, on the management of ... 365
Fractures; spasm of the limb ... 369
Fractures, extension for ... 371
Fractures, splints for ... 372
Fractures of the leg ... 373
Fractures, Mr. Radley on ... 375
Fractures, mischiefs of splints ... 375
Gall-stones, symptoms and treatment, 251
Gall-stones, cases of ... 251
Ganglionic tumor, cause of ... 47
Ganglionic nerves, M. Brachet on the, 57
Ganglionic system, general functions of 60
Ganglionic system, special functions of 67
Ganglionic system, M. Brachet on .. 414
Ganglionic system, M. Lobstein on .. 414
Ganglionic nerves, influence of, on the stomach ... 414
Gangrene, white, of the skin ... 341
Gastrodynia; disposition to suicide .. 169
Gonorrhœa and syphilis, supposed identity of ... 307
Gonorrhœa, supposed to be a pustule, 307
Gonorrhœa, occasions abscess of penis, 308
Gonorrhœa, origin of ... 309
Gonorrhœa, from ulcer of the orifice of the urethra ... 310
Gonorrhœa and syphilis constantly co-exist in prostitutes ... 312
Gonorrhœa, secondary symptoms from 313
Gonorrhœa, with syphilitic sores in the urethra ... 315
Gonorrhœa, Mr H. J. Johnson on .. 488
Gonorrhœa, retention of urine from.. 554
Gonorrhœal secondary symptoms.... 316
Gout, on the proximate cause of .... 540
Gout, etiology of ... 541
Gout, does it occasion longevity?.... 542
Great sympathetic, influence of, on the heart ... 73
Grippe, epidemics of the ... 489
Guthrie, Mr. on removing large calculi 287
Gymnotus electricus ... 93
Hag, singular means of escape of the, 92
Headache cured by tonics ... 476
Heart, M. Bouillaud on diseases of the, 353
Heart, inflammation of the lining membrane of ... 354
Heart, influence of affections of its right ventricle on the lungs ... 513
Hemiplegia—peculiar case ... 569
Hiccough, cured by a blister ... 192
Hohl, Dr. on auscultation of pregnancy 220
Horse, cases of palsy in the ... 478
Hospital physicians and surgeons, election of, in France ... 76
Houston, Dr. on fractures ... 365
Howard, Mr. his account of the epidemic scarlatina in Yorkshire .... 292
Hughes, Dr. on pericarditis ... 547
Humboldt's divisions of temperatures, 393
Hydatids in bone ... 15
Hydatids of the bladder ... 255
Hydriodate of potass, Dr. Clendinning's observations on ... 241
Hydriodate of potass, in uterine disease 241
Hydriodate of potass in nodes ... 243
Hydriodate of potass in rheumatism.. 243
Hyd. of potass in syphilitic eruptions, 262
Hyd. of potass for roseola syphilitica.. 262
Hydriodate of potass for purpura ditto 264
Hydriodate of potass for rupia ... 264
Hydriodate of potass for lichen ... 265
Hydrocele, treated by the seton ... 283
Hydrocele of the neck, Mr. Cooper's case of ... 575
Hydrocele of the neck, occasional dangers of seton ... 576
Hydrocephalus acutus from scarlatina 570
Hydrocephalus chronicus ... 571
Hypertrophy of bone ... 1
Hypertrophy of bone, varieties of.... 2
Hypertrophy of brain by Dr. Sims .. 117
Hypertrophy and atrophy of brain .. 125
Hypertrophy of the brain—enteritis.. 126
Hypertrophy of the brain—extravasation of blood ... 126
Hypertrophy of the brain—convulsions. &c. ... 127
Hypertrophy of the brain, pulmonary tubercles ... 127
Hypertrophy of one hemisphere—apoplectic cysts ... 128
Hypertrophy of brain, remarks on .. 129
Hypertrophy, Dr. Carswell on ... 345
Hypertrophy, origin and causes of .. 346
Hypertrophy, physical characters of.. 351
Hypertrophy of heart, influence of, on cerebral disease ... 507
Hypertrophy, cardiac, effects on mind 509
Hypertrophy, cardiac, occasions apoplexy ... 510
Hypertrophy, M. Bouillaud on ... 511
Hypertrophy, M. Rochoux on ... 511
Hypertrophy, Dr. Copland on ... 512
Hypertrophy, Dr. Hope on ... 512
Hysterical pains, amputation for .... 45
Inflammation of the ligaments ... 19
Influenza, epidemics of ... 498
Influenza in 1743 ... 499
Influenza in 1775 ... 500
Influenza in 1830 ... 500
Injuries of the head, cases of ... 274
Instinct ... 84
Instinct, proximate cause of ... 88
Insanity, partial without fever ... 530
Insurance offices ... 594
Intermittent fever, bloodletting in.... 189
Internal hæmorrhage, tendency to, in ascites ... 115
Intestinal irritation, convulsions from, 173
Iodine, salivation from ... 203

Irritation simulating inflammation .. 163
Isothermal zones . ............... 393
Jennings, Dr. on phlegmatia dolens.. 469
Johnson, Dr. his case of disease of stomach and colon ................ 184
Johnson, Dr. his case of remarkable disease of brain ................. 202
Johnson, Dr. his case of cessation of pulse in many large arteries ...... 451
Johnson, Mr. H. J. on gonorrhœa.... 488
Johnson, Mr. H. J. on inflamed absorbents of penis .................. 489
Johnson, Mr. H. J. on abscess of perineum ....................... 490
Johnson, Mr. H. J. inflammation of the prostate gland.................. 492
Johnson, Dr. on disease of heart, &c.. 588
Joints ........................... 15
Joints, synarthroses of ............ 16
Joints, diarthroses of .............. 17
Joints, neuralgic affection of........ 19
Joints, diseased, excision of ........ 25
Judd, Mr. on urethritis and syphilis.. 305
Key, Mr. on ulceration of cartilage .. 443
Kirby, Mr. on the wisdom of God in the creation of animals............... 79
Kyan's patent for dry-rot ........... 521
Labour, premature, induction of .... 178
Labour, change of auscultic sounds in 224
Lactation, influence of, on population, 440
Lactation, period of ............... 441
Lallemand, M. on erectile tumors.... 504
Language, impairment of the faculty of 207
Lee on medical institutions of France, 75
Lee on fibro-calcareous tumors in the ute[illegible]s ....................... 96
Lee, Dr. on the fœtal kidney........ 453
Life, chances of .................. 461
Ligaments, inflammation of ........ 19
Lithotomy, second. hæmorrhage from, 199
Lithotomy, Mr. Lizars' anomalous case 456
Liver, case of rupture of .......... 296
Liver, Mr. Conwell on diseases of the, 378
Liver, causes of disease of.......... 379
Liver, symptoms of diseases of...... 380
Liver, terminations of diseases of.... 382
Liver, chronic inflammation of...... 384
Lives of literary persons............ 462
Lobstein, M. on the sympathetic .... 429
Loudon, Dr. on population and sustenance ......................... 439
Lungs and heart, Dr. Davies on diseases of the..................... 353
Lymphatics, M. Velpeau on diseases of 244
Lymphatics, action of the, in disease, 245
Lymphatics, inflammation of the.... 246
Macilwain, Mr. on sympathy ....... 401
Malformations of the internal ear, cases and dissections of ............... 194
Malignant growth of bone.......... 12
Man, functions and instincts of...... 363
Marshall, Dr. on spinal irritation .... 146
Marshall, Dr. his reclamation ...... 587
Mart's observations on nerv. diseases, 49
Matter, divisibility of.............. 388
Matter, molecular constitution of.... 389
Mayo's outlines of pathology ....... 1
Mayo, Mr. his outlines of pathology.. 328
Maxillary bone, superior, removal of. By Dr. Tuthill.................. 476
Maxillary bone, superior, Mr. Syme on 558
Maxillary bone excised, cases of .... 558
Maxillary bone, cysts in. .......... 559
Medical charities in Ireland, inquiry into 34
Medical institutions of France, &c. Mr. Lee on ..................... 75
Medical profession, remuneration of, on the continent .................. 76
Medical politics .................. 590
Medical reform ................... 596
Medico-chirurgical Transact. vol. xix. 94
Medullary sarcoma of face—operation unsuccessful ................ 565
Membranes, serous effusion from, by Dr. Sims ...................... 117
Memoirs of Royal Academy of Medicine 517
Mental derangement, cases of, by Dr. Seymour ...................... 117
Meteorology, Dr. Prout on ......... 391
Metropolitan university............ 596
Middlemore, Mr. on pterygium ...... 179
Milky state of blood, cases of........ 229
Mollities ossium, case of .......... 6
Mollities ossium, case of .......... 254
Monoculus polyphemus............ 81
Monstrosities, general view of ..... 209
Monstrosities, divisions of.......... 209
Monstrosities, laws of ............. 210
Monstrosities, male and female...... 211
Monstrosities, with deficient parts.... 212
Monstrosities, theory of M. Serres ... 213
Morphia in mania ................ 141
Morrison's pills, death from ........ 290
Muscles, affections of.............. 330
Muscles, hypertrophy and atrophy of, 331
Muscles, chronic inflammation of. .. 332
Muscles, entozoa found in.......... 334
Nakra, the ...................... 477
Neck of the femur, fracture of ...... 102
Neck, encysted tumor of, cured by seton 560
Necrosis, trephining for............ 8
Neuralgia ....................... 56
Nerves, inflammatory action in...... 41
Nervous tissue, Mr. Travers on lesions 38
Nervous diseases, observation on by George Mart ................... 49
Nervous indigestion .............. 54
Nervous system, influence of, on large intestine ................... 425
Nervous system, Mr. Swan's plates of the comparative anatomy of...... 435
Nitrate of silver to small-pox pustules 249
Nitric acid poured into ear, death from 562
Northwoods lunatic asylum ........ 605

Notencephale, case of............... 484
Obliteration of aorta, case of........ 217
Obliteration of vena cava, cases of .. 219
Operating on tumors, injurious effects, 45
Organic lesions in the brain .... ... 40
Osborne, Dr. on dropsies connected with suppressed perspiration...... 158
Ovarian dropsy cured by a blow .... 482
Palsy in the horse, Mr. Youatt on ... 478
Par vagum; M. Brachet's experiments 415
Par vagum, results of division of .... 421
Par vagum, galvanic experiments on, 422
Par vagum, influence of, on digestion, 423
Paracentesis thoracis, for hydrothorax, 162
Paralysis agitans.................. 52
Paralysis from want of stimulus .... 554
Paralytic diseases ................ 49
Paraplegia ...................... 50
Parker, Mr. on diseases of the stomach 167
Pathological knowledge, diffusion of in France................. ... 78
Pathology, Mr. Mayo's outlines of ... 1
Pathology, Mr. Mayo's outlines of.... 328
Pathology, comparative ............ 478
Pauper practice .................. 591
Perforation of stomach not fatal.... 497
Pericardial exudation, cases of ...... 454
Pericarditis, Dr. Hughes on ... .... 547
Pericarditis, diagnosis of .......... 547
Pericarditis, from rheumatism ...... 550
Pericarditis, conclusions respecting .. 551
Perineum, on abscess of the ........ 490
Peritoneum, morbid conditions of.... 113
Peritonitis, chronic, Dr. Johnson's case 161
Phelan's inquiry into medical charities in Ireland .................... 34
Phlegmatia dolens, pathology of .... 469
Phosphorescence of organized bodies, 486
Phosphorescence of plants.......... 487
Phosphorescence of lampyrus ....... 487
Phosphorescence, causes of ........ 488
Phrenological quacks.............. 208
Placentary bruit, account of the .... 225
Plague, contagion of.............. 544
Pneumo-gastric nerves, influence of on the heart... ................ 71
Pneumo-gastric nerves, division of the 72
Population and sustenance, Dr. Loudon 439
Population, annual increase of ...... 439
Population, influence of lactation on, 440
Practical men—who are they?...... 257
Pregnancy, auscultic signs of ...... 220
Premature labour, induction of...... 178
Prostate, inflammation of the ...... 492
Prout, Dr. his Bridgewater treatise .. 385
Provincial medical education........ 173
Pterygium, Mr. Middlemore on ..... 179
Pterygium, forms of .............. 180
Pterygium, treatment of .......... 181
Pulmonary phlebitis, case of........ 94
Pulmonary apoplexy, from disease of auriculo-ventricular aperture .... 172
Pulmonary apoplexy, cases of ...... 171
Pulmonary exhalation, Tiedemann on, 468
Pulmonary circulation, how influenced by lesions of right ventricle ...... 513
Pulmonary apoplexy, cases of ...... 516
Pulse of the fœtus—tables of the .... 228
Pulse, cases of cessation in large arteries 451
Puncture of bladder above pubes .... 166
Purpura and carditis—death from .. 291
Purulent deposits ................ 31
Purulent depôts, Dr. Nasse on ...... 531
Purulent depôts in lungs .......... 532
Purulent depôts in liver............ 533
Purulent depôts, symptoms of ...... 535
Purulent depôts, causes of.......... 536
Purulent depôts, affections of veins and lymphatics in .............. 537
Purulent depôts, various hypotheses, 536
Pus, mode of production of ........ 28
Pus, process of suppuration of ...... 28
Pus, general properties of.......... 33
Ramollissement of the brain, cure of, 117
Ramollissement of brain, Dr. Sims on, 135
Ramollissement of the brain, cases of, 136
Ramollissement of the brain, remarks, 140
Reclamation, Dr. Marshall's........ 587
Rectum, Sir B. Brodie on ulcer of.... 281
Rectum, Sir B. Brodie on stricture of, 282
Reform, medical.................. 596
Reptiles, Mr. Kirby's account of .... 358
Retention of urine from gonorrhœa.. 554
Rheumatism, Dr. Wilson on........ 409
Rheumatism, pathology of ........ 469
Ribs, case of inflammation of ...... 7
Saliva, state of, in affections of the stomach ... ................ .. 231
Saliva, acidity of, morbid .......... 232
Saliva, usually alkaline ............ 232
Saliva, in various diseases......... 233
Salivation from iodine ............ 203
Scandinavian medicine ............ 189
Scarlatina maligna, epidemic of, in Yorkshire ... ................ 292
Scarlet fever, Dr. Sandwith on ...... 175
Schools of London, present state of .. 276
Secondary abscesses, Dr. Nasse on . 531
Serous effusion, and its connexion with apoplexy.................. 117
Serous effusion, with no cerebral symptoms .................... 119
Serous effusion, with apoplectic cysts, 121
Serous effusion, with extravasation of blood ...................... 121
Serous effusion, with loaded blood-vessels...................... 122
Serres, M. his theory of monstrosities, 213
Seton, Mr. Green on the, for hydrocele, 283
Seymour, Dr. his cases of mania . .. 141
Sheet lead for ulcers .............. 501
Sims, Dr. on diseases of the brain .. 117
Skin, white gangrene of the ........ 241
Skin, hypertrophy of ............ 342

Small-pox at Naples .............. 234
Small pox in London, in 1834 ...... 235
Small-pox after vaccination—Dr. Gregory's observations.............. 236
Small-pox in Germany ............ 237
Small-pox at the Chelsea Asylum .. 239
Small-pox in Christ's Hospital..... 239
Small-pox at Cricklade ............ 239
Small-pox pustules, local applications, 249
Smith, Mr. his surgical report ...... 197
Spinal diseases, Dr. Enz on ........ 523
Spinal diseases, colic, &c. from....... 525
Spinal diseases, cough, &c. from .... 527
Spinal disease, convulsions from .... 529
Spinal disease, partial insanity from.. 530
Spinal irritation, Dr. Marshall on.... 146
Spinal irritation, supposed cases of .. 147
Spinal irritation, simulating disease of the heart....................... 152
Spinal irritation, simulating disease of the lungs....................... 154
Spinal irritation, simulating asthma.. 156
Spinal irritation, simulating diabetes.. 157
Spinal marrow, concussion of . .... 43
Spinal marrow, influence of, on heart, 70
Sphincter ani, Sir B. Brodie on spasmodic contraction of the ........ 281
Spleen, Mr. Twining on diseases of .. 449
Staphyloma, cured by excision of the cornea.......................... 543
Statistics, Dr. Graves on .......... 461
Stercoraceous vomiting for 18 months —Mr. Dickson's case......... ... 470
Stomach and colon, Dr. Johnson's case of remarkable disease of...... 184
Stomach—source of its contractions.. 418
Stomach, perforation of, not fatal.... 497
Suicide—desperate wound of neck .. 187
Suicides, in France, Ireland, &c. .... 463
Suppuration, Mr. Key's notions on .. 444
Suppuration; agency of absorption.. 446
Surgical disease, difference in treatment of, in France.............. 78
Swan's, Mr. comparative anatomy of the nerves ...................... 435
Sweats, blue, case of .............. 343
Sympathetic system of nerves, M. Lobstein on the ................. 429
Sympathy, Mr. Macilwain on ...... 401
Sympathy, natural or morbid ..... 402
Sympathy of tissues .............. 403
Sympathy, Dr. Wilson ............ 405
Synovial membrane, inflammation of, 21
Synovial membrane, affections of.... 25
Syphilitic sores, tabular view of .... 325
Syphilis, Mr. Judd on ............ 305
Syphilis among the Jews .......... 317
Syphilis, spontaneous origin of...... 317
Syphilis, from condylomata ........ 318
Syphilis, plurality of poisons........ 319
Syphilis; re-ulceration of the cicatrix, 322
Syphilitic affections of the testis .... 206
Syphilitic eruptions, treated with hydriodate of potass .............. 262
Temperature of different parts of the body in disease ................ 456
Temperature of various portions of the globe ........................ 392
Temperature, variations of ........ 398
Testis, Dr. Cusack on venereal affections of the.................. 205
Thorburn, Dr. his bedside medicine .. 177
Thurnam, Mr. his dissection of a malformation of the internal ear...... 196
Tic douloureux .................... 55
Tiedemann, on the phosphorescence of living bodies .................. 486
Timber—dry rot—Kyan's patent .... 521
Torbet, Mr. his cases of neuralgia.... 163
Torpedo, electricity of the.......... 92
Torsion of arteries, M. Clot Bey on.. 518
Transcendentalisms ............ .. 467
Transposition of the viscera, case of.. 215
Travers, Mr. on constitutional irritation 37
Travers, Mr. his case of absorption of the cranium .......... ..... 285
Treatment of corns and bunions .... 585
Trephining for necrosis ............ 8
Tubercles in the monkey .......... 467
Tumors of nerves, origin of ........ 46
Tumor, aneurysmal, in the orbit .... 584
Turner, Professor, death of ........ 301
Twining, Mr. on diseases of spleen .. 449
Tyerman's Mr. case of wound of neck, 187
Ulceration of cartilage, Mr. Key on.. 443
Ulcers, treated by sheet lead........ 501
Ununited fracture of humerus, cured by excision . .................... 561
Upper jaw, right, removed ........ 476
Urethra, cases of syphilitic sores in the, 315
Urethritis and syphilis, Mr. Judd on.. 305
Urethritis and syphilis, identity of ?.. 306
Urethritis and syphilis, comparison of, 307
Uterus, laceration of peritoneal coat of, 95
Uterus, fibro-calcareous tumors in .. 96
Uterus, case of total loss of . ...... 482
Velpeau, M. on diseases of lymphatics, 244
Vena cava, case of obliteration of.... 219
Venereal affections of the testis .... 205
Viscera, transposed, case of ........ 215
Voyage round the world, Dr. Wilson's, 143
Warren, Dr. sketch of .. .......... 300
Warty tumors in cicatrices, cases of.. 98
Warty tumors, characters of........ 99
Warty tumors, knife preferable to caustics in ..................... 101
Wilson's, Dr. voyage round the world, 143
Wilson, Dr. on sympathy .......... 405
Wound of neck, desperate.......... 187
Wound of scalp, with symptoms of phrenitis........................ 274
Wounded arteries, cases of ........ 551
Wounded brachial in venæsection... 551
Wounded radial .................. 552

